# The role of early venous duplex screening in improving patient safety indicator-12 (PSI-12) classification accuracy: a retrospective cohort study

**DOI:** 10.1186/s13037-026-00489-9

**Published:** 2026-06-08

**Authors:** Adam S. Surti, Robert T. Matthews, Oana Popescu, Malak Bentaleb, Rodrigo F. Alban

**Affiliations:** 1 Department of Surgery, Cedars-Sinai Medical Center, 8700 Beverly Blvd, 8215NT, Los Angeles, CA 90048 USA; 2 College of Medicine and Health Sciences, Khalifa University, Abu Dhabi, United Arab Emirates

**Keywords:** Venous thromboembolism (VTE), Venous duplex ultrasound, Patient safety indicator-12 (PSI-12), Misclassification, Patient safety

## Abstract

**Background:**

Venous thromboembolism (VTE) is a major cause of morbidity among surgical patients. Patient Safety Indicator 12 (PSI-12) identifies perioperative VTE events. This study evaluates whether early admission duplex ultrasound identifies VTEs associated with PSI-12 coding and assesses PSI-12 classification accuracy.

**Methods:**

A single-center retrospective analysis was conducted on surgical patients with a venous duplex ultrasound within 48 h of admission at a large academic medical center (2013–2024). Two cohorts were analyzed: (1) all surgical patients with early duplex imaging, (2) PSI-12 positive elective surgical patients with early duplex imaging. The primary outcome was the prevalence of pre-existing DVT identified within 48 h among PSI-12 positive patients. Diagnostic accuracy of early abnormal duplex findings for predicting PSI-12 classification was assessed using sensitivity, specificity, positive predictive value (PPV), and negative predictive value (NPV).

**Results:**

Among all 10,498 surgical patients, 10.6% had positive duplex findings for DVT. PSI-12 positive patients had double the prevalence compared to PSI-12 negatives (20.9% vs. 10.4%, *p* < 0.001). In PSI-12 positive elective cases, 10.7% of preoperative duplex studies identified a DVT. Misclassification, defined as PSI-12 positive admissions without imaging-confirmed DVT or pulmonary embolism, occurred in 8.7% of elective cases. Overall, 36.7% of PSI-12 positive patients had abnormal early duplex findings. Early duplex demonstrated limited sensitivity but high specificity (sensitivity 36.7%, specificity 81.7%, PPV 3.7%, NPV 98.5%).

**Conclusions:**

Our findings suggest possible pre-existing disease rather than hospital-acquired VTE in PSI-12 positive patients. Incorporating admission duplex studies could improve PSI-12 classification accuracy, reduce costs, and enhance patient safety.

## Background

Perioperative venous thromboembolism (VTE), encompassing deep vein thrombosis (DVT) and pulmonary embolism (PE), is a significant source of morbidity and mortality in hospitalized patients. The incidence of VTE varies based on surgery type and patient-specific risk factors, with particularly high rates observed in patients undergoing major surgery (15–40%), hip and knee arthroplasty (40–60%), and trauma operations (60–80%) [1]. Preoperative duplex screening in asymptomatic high-risk cancer patients has revealed a VTE prevalence of 10% [2], underscoring the critical importance of accurate perioperative risk assessment and preventive strategies in surgical care.

To address these challenges and track hospital quality improvement, the Agency for Healthcare Research and Quality (AHRQ) developed the Patient Safety Indicators (PSIs) in 2003. These metrics are designed to identify potentially avoidable adverse events occurring during hospital stays, promote quality improvement initiatives, rank surgical program performance, and impose financial penalties on hospitals [3, 4]. PSI-12 is one of these metrics that specifically focuses on perioperative DVT and PE events, which are associated with an estimated additional cost of $17,367 per event and a 4.3% increase in excess mortality [5].PSI-12 events are determined from administrative ICD-10 codes documented by hospital coders, capturing any DVT or PE events during the hospital admission as hospital acquired and thus perioperative. PSI-12 is calculated using administrative discharge data and is structured as a numerator–denominator-based quality measure, in which the denominator defines the population at risk and the numerator captures qualifying postoperative VTE. A hospitalization enters the denominator if the patient is ≥ 18 years old with a qualifying surgical MS-DRG and an operating room procedure as defined by AHRQ specifications [6]. The numerator includes hospitalizations with secondary ICD-10 diagnosis code of proximal DVT, or pulmonary embolism assigned during the hospitalization only when the diagnosis is not marked Present on Admission (POA) [6]. Cases are excluded if VTE is POA, if certain conditions or procedures are present (e.g., ECMO, HIT, early IVC filter), or if key data are missing [6].

Recently, however, the validity and accuracy of these PSIs have been increasingly questioned. Studies suggest limitations in its ability to accurately identify in-hospital complications and reflect true quality of care [7]. A study published in the *Journal of Hospital Medicine* found that PSI-12 rarely identifies problems with care quality [8], and another study in the *Journal of Vascular Surgery* found that PSIs are inferior to metrics like the Vascular Quality Initiative (VQI) and National Surgical Quality Improvement Program (NSQIP) in identifying complications [9]. Additional research has demonstrated these indicators are unable to capture clinically relevant complications [10, 11], and that these quality evaluations often rely on inaccurate medical record documentation [12] or miscoded diagnoses and inaccurate ICD-10 codes [13]. Further, unless explicitly documented as Present on Admission (POA), any pre-existing DVT or PEs found during a hospital admission can be classified as a PSI-12 positive admission. Critically, because PSI-12 classifications rely solely on coded diagnoses without requiring clinical validation or imaging interpretation review, pre-existing DVTs may be misclassified as hospital-acquired events. This supports the need for ongoing evaluation and refinement of these indicators.

Given these challenges, this study aims to evaluate whether early admission duplex ultrasound in surgical patients can identify pre-existing DVTs among PSI-12 coded surgical patients, and to assess the accuracy of PSI-12 coding by quantifying the rate of misclassification. By examining the relationship between early duplex ultrasound and PSI-12 classification, we seek to assess the effectiveness of current screening protocols and propose improvements in perioperative VTE detection and prevention strategies.

## Methods

### Study design and population

This study data was generated from a quality improvement database at Cedars-Sinai Medical Center that contained de-identified data. Analysis of deidentified data generated for purposes other than research is determined to meet “Not Regulated” status by the Institutional Review Board (IRB) of Cedars-Sinai Medical Center and ensures compliance with ethical standards in research.

We conducted a single-center retrospective analysis of all adults (≥ 18 years) patients who underwent a surgical procedure and had a venous duplex ultrasound study performed within the first 48 h of their admission at a large quaternary, academic medical center and Level 1 trauma hospital from 2013 to 2024. The 48 h threshold was selected based on prior studies which treated VTE diagnosed within 48 h of admission as likely present on admission rather than hospital acquired [14–17].

At our institution, routine venous duplex screening within 48 h of admission is frequently performed for high-risk patients (e.g. oncologic or trauma operations) but is not universally protocolized. In our study, the most common indications reported for duplex ultrasound evaluation were “swelling”, “history of thrombus”, and “surveillance”. Ordering typically reflects surgeon or admitting physician discretion based on patient risk profiles.

Demographic and clinical characteristics collected include age, sex, admission diagnoses (diabetes, hypertension, congestive heart failure, etc.) and Elixhauser and Charlson comorbidity indices.

### Duplex interpretation

All duplex ultrasound impressions were analyzed and reviewed by two independent reviewers using custom text-mining software that flagged key terms such as “thrombus”, “acute”, “chronic”, “reflux”, etc, enabling identification of patients with positive or abnormal findings. Impressions with findings of acute basilic, cephalic, and external jugular were classified as acute superficial upper thrombus. Impressions with findings of acute brachial, axillary, subclavian, innominate and internal jugular were classified as acute deep upper thrombus. Impressions with findings of acute great saphenous and small saphenous were classified as acute superficial lower thrombus. Impressions with findings of acute soleal, gastrocnemius, peroneal, anterior or posterior tibial, popliteal, femoral, deep femoral or iliac were classified as acute deep lower thrombus. Chronic thrombi and venous reflux were recorded separately as abnormal findings. Abnormal findings were analyzed because they represent potential precursors to acute thrombus formation later during hospitalization, which, if not present within the first 48 hours, could ultimately result in new DVTs classified as PSI-12 positive admissions. The prevalence of venous thrombi was calculated as the proportion of patients with acute deep vein thrombosis.

### PSI-12 classification

PSI-12 status was determined using administrative ICD-10 diagnosis codes and Present-on-Admission (POA) indicators according to AHRQ specifications. Patients with postoperative proximal DVT or pulmonary embolism not marked POA were classified as PSI-12 positive.

### Primary outcome

The primary outcome of this study is to determine the prevalence of pre-existing DVT in patients classified as PSI-12 positive, using duplex ultrasound performed within 48 h of admission.

### Cohort definitions

The study cohort was divided into two subsets:

1. All Surgical Patients with Early Venous Duplex Ultrasound

The first cohort included all patients who underwent elective or emergent surgery and had received a venous duplex ultrasound performed within the first 48 h of admission. Venous duplex ultrasound impressions were analyzed to identify positive findings, including acute superficial or deep thrombus. Patients were stratified by PSI-12 status and prevalence of early acute thrombus was compared. Comparisons between these two groups were performed using a two-sample unpaired z-test, with statistical significance defined as *p* < 0.05.

2. Elective Surgical Patients with PSI-12 Hospital Admissions

The second cohort consisted of patients who underwent solely elective surgical procedures and were classified as PSI-12 positive admissions. To further analyze the timing and potential origin of thrombi, this cohort was divided into preoperative and postoperative groups based on the timing of their duplex ultrasound. All preoperative ultrasound scans were performed within 48 hours of admission and prior to surgical incision. Additionally, to evaluate PSI-12 coding accuracy, all patient full charts in this cohort were reviewed individually by two independent reviewers to further assess PSI-12 classification accuracy – as individual chart review would allow for capturing if any DVT or PE was present in their hospital admission after the initial 48 hours.

### Secondary outcome

The secondary outcome of this study was to determine misclassification of patients within the elective cohort. Any patient characterized as a PSI-12 positive admission without evidence of a DVT or PE in that admission (duplex ultrasound with positive acute deep vein thrombus or CT chest with findings of pulmonary embolism) was characterized as misclassified.

### Statistical analysis

Data were summarized using mean ± standard deviation for continuous variables and proportions for categorical variables. Comparisons between PSI-12 positive and negative groups were performed using an unpaired, two-tailed z-test and Mann-Whitney U test. Statistical significance was defined as *p* < 0.05. All analyses were conducted using R software 4.4.2 GUI 1.81 Big Sur ARM build. Diagnostic accuracy of early abnormal venous duplex findings for predicting PSI-12 positivity was assessed using sensitivity, specificity, positive predictive value (PPV), and negative predictive value (NPV), with PSI-12 status serving as the reference standard.

## Results

A total of 10,498 surgical patients who underwent venous duplex ultrasounds within 48 h of admission were included in the analysis. Of these, 196 (1.9%) patients were classified as PSI-12 positive, and 10,302 were PSI-12 negative.

The mean age across the entire cohort was 65 years, with no significant differences between PSI-12 positive and negative groups. Gender distribution was similar, with 47.5% female in both groups. However, ethnic disparities were noted, with a higher proportion of White patients in the PSI-12 positive group compared to the PSI-12 negative group (63% vs. 56%, *p* = 0.04), and a lower proportion of Asian patients in the PSI-12 positive group (2.5% vs. 6.2%, *p* = 0.03) (Table 1).

**Table 1:** Demographic information

Demographics	PSI Positive *N* = 196	PSI Negative *N* = 10,302	Total *N* = 10,498	*P* value
**Age at admission**				
Average (Year, +/- SD)	63.3, +/-15.3	65.1, +/- 16.5	65.0, +/- 16.0	0.085
**BMI > 30 on admission**				P value
Prevalence (n, %)	59, 29.9%	3246, 31.5%	3305, 31.5%	0.631
**Gender (Female)**				
Prevalence (n, %)	96, 47.9%	4823, 46.8%	4919, 46.6%	0.757
**Ethnicity**				
**Asian**				
Prevalence (n, %)	5, 2.6%	641, 6.1%	646, 6.2%	0.041
**African American**				
Prevalence (n, %)	27, 13.8%	1769, 16.9	1796, 17.1%	0.250
**Hispanic**				
**P**revalence (n, %)	27, 13.8%	1509, 14.4%	1536, 14.7%	0.810
**White**				
Prevalence (n, %)	124, 63.3%	5849, 56.0%	5973, 56.9%	0.041
**Other**				
Prevalence (n, %)	13, 6.6%	685, 6.5%	698, 6.6%	0.952

Demographic information of all surgical patients with venous duplex performed in the first 48 h of admission grouped by PSI–12 designation. Ages were compared with Mann-Whitney U test and prevalences were compared with unpaired two sample z test. P value of < 0.05 was used for significance

Comorbidity indices revealed a sicker population among PSI-12 positive patients, with significantly higher rates of congestive heart failure (49.7% vs. 41.6%, *p* = 0.03) and electrolyte disorders (74.6% vs. 66%, *p* = 0.01). Additionally, conditions such as paralysis (10.2% vs. 6.4%, *p* = 0.03) and metastatic cancer (21.3% vs. 16.2%, *p* = 0.05) were more prevalent in the PSI-12 positive group. The Elixhauser comorbidity indices also indicated a sicker PSI-12 population (28.4 +/- 18.3 vs. 24.1 +/- 17.3, *p* < 0.001) (Table 2).

**Table 2:** Comorbidity information

Comorbidities	PSI Positive *N* = 196	PSI Negative *N* = 10,302	Total *N* = 10,498	*P* value
**Any malignancy**				
Prevalence (n, %)	68, 34.5%	3198, 30.9%	3266, 31.1%	0.280
**Cerebrovascular disease**				
Prevalence (n, %)	9, 4.6%	651, 6.3%	660, 6.3%	0.332
**Coagulopathy**				
Prevalence (n, %)	77, 39.1%	3285, 31.8%	3362, 32.0%	0.105
**Congestive heart failure**				
Prevalence (n, %)	82, 41.6%	5125, 49.6%	5207, 49.6%	0.026
**Diabetes**				
Prevalence (n, %)	60, 30.5%	3738, 36.2%	3798, 36.2%	0.220
**Fluid and electrolyte disorders**				
Prevalence (n, %)	147, 74.6%	6804, 65.8%	6951, 66.2%	0.010
**Hypertension**				
Prevalence (n, %)	160, 81.2%	8088, 78.2%	8248, 78.6%	0.379
**Myocardial infarction**				
Prevalence (n, %)	43, 21.8%	2637, 25.5%	2680, 25.5%	0.238
**Metastatic Cancer**				
Prevalence (n, %)	42, 21.3%	1670, 16.2%	1712, 16.3%	0.055
**Obesity**				
Prevalence (n, %)	60, 30.5%	3155, 30.5%	3215, 30.6%	0.989
**Paralysis**				
Prevalence (n, %)	20, 10.2%	654, 6.3%	674, 6.4%	0.027
**Solid tumor without metastasis**				
Prevalence (n, %)	65, 33.0%	2793, 27.0%	2858, 27.2%	0.061
**Weight loss**				
Prevalence (n, %)	40, 20.3%	1552, 15.0%	1592, 15.2%	0.049
**ICD 10 Exclusion in admission diagnosis**				
Prevalence (n, %)	88, 44.9%	1164, 11.3%	1252, 12.2%	< 0.001
**Charlson Score**				
Average (Score, +/- SD)	4.7, +/- 3.8	4.4, +/- 3.6	4.4, +/- 3.6	0.452
**Elixhauser IP Mortality Index**				
Average (Index, +/- SD)	28.4 +/- 18.3	24.1 +/- 17.3	24.2, +/- 17.3	0.009

Comorbidity information of all surgical patients with venous duplex performed in the first 48 h of admission grouped by PSI – 12 designation. Indexes were compared with Mann-Whitney U test and prevalences were compared with unpaired two sample z test. P value of < 0.05 was used for significance

PSI-12 positive patients had significantly longer operations, with 28.8% lasting over three hours compared to 14.7% of PSI-12 negative patients (*p* < 0.01). Length of hospital stay was also notably longer in PSI-12 positive patients, with 81.1% staying over seven days compared to 53.2% of PSI-12 negative patients (*p* < 0.01) (Table 3).

**Table 3:** Procedure and admission time

Procedure and Admission Time	PSI Positive *N* = 196	PSI Negative *N* = 10,302	Total *N* = 10,498	*P* value
Length of Operation (> 3 h)				
Prevalence (n, %)	56, 28.8%	1512, 14.7%	1568, 14.9%	< 0.001
Length of Admission (> 7 days)				
Prevalence (n, %)	159, 81.1%	3246, 31.5%	3405, 32.4%	< 0.001

Procedure and admission time for patients were compared using unpaired two sample z test. P value of < 0.05 was used for significance

Among all surgical patients, 10.6% had positive duplex studies within the first 48 h of admission. In the PSI-12 positive group, 20.9% had positive duplex findings compared to 10.4% in the PSI-12 negative group (*p* < 0.01), indicating a significantly higher prevalence of early thrombi in PSI-12 patients. Notably, in this PSI-12 positive group, 140 out of 196 duplex ultrasounds (71.4%) were performed preoperatively. Superficial thrombi were also more frequent in PSI-12 positive patients (9.6% vs. 3.7%, *p* < 0.01). In total, 36.7% of the PSI-12 positive group had any abnormality when compared to the 18.3% in the PSI-12 negative group (*p* < 0.001) (Table 4). These abnormal findings represent early venous pathology that may progress to acute thrombus formation later in the hospitalization.

**Table 4:** Ultrasound findings

Ultrasound Findings	PSI Positive *N* = 196	PSI Negative *N* = 10,302	Total *N* = 10,498	*P* value
**Venous Reflux**				
Prevalence (n, %)	2, 10.2%	268, 2.6%	270, 2.5%	0.166
**Chronic Thrombus**				
Prevalence (n, %)	5, 2.6%	399, 3.9%	404, 3.8%	0.341
**Acute superficial upper venous thrombus**				
Prevalence (n, %)	14, 7.1%	313, 3.0%	327, 3.1%	0.001
**Acute superficial lower venous thrombus**				
**P**revalence (n, %)	5, 2.6%	70, 0.7%	75, 0.7%	0.002
**Acute deep upper venous thrombus**				
Prevalence (n, %)	2, 1.0%	96, 0.9%	98, 0.9%	0.898
**Acute deep lower venous thrombus**				
Prevalence (n, %)	39, 19.9%	980, 9.5%	1019, 9.7%	< 0.001
**Acute superficial venous thrombus**				
Prevalence (n, %)	19, 9.6%	383, 3.7%	402, 3.5%	< 0.001
**Acute deep venous thrombus**				
Prevalence (n, %)	41, 20.8%	1076, 10.4%	1117, 10.6%	< 0.001
**Total Abnormal**				
Prevalence (n, %)	72, 36.7%	1881, 18.3%	1953, 18.6%	< 0.001

Ultrasound findings of all surgical patients with venous duplex performed in the first 48 h of admission grouped by PSI–12 designations. Prevalences were compared with unpaired two sample z test. P value of < 0.05 was used for significance

In the elective cohort, 56 out of 104 duplex ultrasounds were performed preoperatively, with 10.7% of these duplex studies identifying DVTs. Additionally, 13.5% of patients with abnormal initial duplex findings (e.g., chronic thrombus, superficial clot, or reflux) later developed a DVT or PE, suggesting that chronic or superficial disease may serve as an indicator for subsequent thrombosis. Misclassification remained a critical issue in the elective cohort, with 8.7% of patients classified as PSI-12 positive having no evidence of DVT or PE found on chart review underscoring the limitations of coding-based classification.

When any abnormality on early duplex ultrasound (acute, superficial, chronic thrombus, or venous reflux) was evaluated as a predictor of PSI-12 classification, diagnostic accuracy was modest. Sensitivity was 36.7%, and specificity was 81.7%. The positive predictive value (PPV) was 3.7%, while the negative predictive value (NPV) was 98.5%, reflecting the low overall prevalence of PSI-12 events in the cohort (Table 5). These findings indicate that while a normal early duplex effectively excludes PSI-12 positivity, abnormal early duplex findings alone are insufficient as a screening tool to predict PSI-12 events.

**Table 5:** Sensitivity, specificity, PPV, and NPV of PSI-12 using early (≤ 48 h) duplex ultrasound

	PSI-12 Positive	PSI-12 Negative	Total
Abnormal Duplex Positive	72	1,885	1,957
Abnormal Duplex Negative	124	8,417	8,541
Total	196	10,302	10,498

All metrics were calculated using standard 2×2 contingency table

## Discussion

This study highlights key insights into the use of PSI-12 metrics in identifying perioperative venous thromboembolism (VTE) and its associated clinical and financial implications. Our findings demonstrate that a significant portion of PSI-12 classified VTE events were likely present at the time of admission rather than hospital-acquired. Among the larger cohort of all surgical patients, PSI-12 positive patients had a significantly higher prevalence of DVTs on early admission duplex compared to PSI-12 negative patients, with nearly 1 in 5 PSI-12 positive patients having an acute DVT identified within 48 h of admission, a rate that was double that observed in PSI-12 negative patients. Importantly, more than 70% of duplex ultrasounds in the PSI-12 positive group were performed preoperatively (performed within 48 h of admission and prior to surgical incision), strongly indicating that many thrombi attributed to postoperative complications may in fact represent pre-existing disease.

Additionally, PSI-12 positive patients had higher rates of superficial thrombi (9.6% vs. 3.7%), further suggesting a potential progression from superficial to deep thrombi or pulmonary embolism over time during hospitalization. The presence of early abnormalities supports the hypothesis that a subset of PSI-12 positive categorized events was a progression of pre-existing venous disease rather than de novo hospital-acquired thrombosis.

These findings suggest early screening for PSI-12 positive admissions could help distinguish pre-existing disease from in-hospital complications if used in select high-risk patient populations. This would reduce morbidity associated with PSI-12 admissions through early treatment initiation and allow for possible more accurate PSI-12 subcategorization of thrombi that may have been pre-existing.

From a diagnostic performance perspective, early duplex abnormalities demonstrated limited sensitivity (36.7%) but good specificity (81.7%) for predicting PSI-12 classification. The positive predictive value was low (3.7%), reflecting the low prevalence of PSI-12 events in the overall surgical population, whereas the negative predictive value was very high (98.5%), indicating that a normal early duplex reliably excludes subsequent PSI-12 classification. This illustrates that while early duplex alone is insufficient as a standalone screening test for PSI-12 classification, it provides clinically meaningful additive information regarding thrombus timing. Early duplex ultrasound should not be used as a universal screening tool to predict PSI-12 events, but rather as a targeted confirmatory tool in high-risk patients where the probability of pre-existing disease is higher.

Among the elective cohort, misclassification remains a major concern in PSI-12 reporting, with nearly 1 in 10 of elective PSI-12 positive cases showing no documented evidence of DVT or PE upon chart review at any point during hospitalization. These errors not only inflate PSI-12 rates but also distort hospital performance metrics, leading to misdirected quality improvement efforts and unjustified financial burdens. Given PSI-12 reliance on administrative coding rather than clinical validation, this further highlights a critical opportunity to improve documentation practices and coding accuracy. In addition, we found a preoperative prevalence of true DVTs in 10.7% of elective cases in our hospital, which aligns with prior studies from our institution [2]. These findings underscore the vulnerability of PSI-12 and the importance of integrating more rigorous clinical reviews into PSI-12 reporting processes.

The financial implications of PSI-12 misclassification are substantial. According to AHRQ estimates, each PSI-12 event adds $17,367 in costs per hospital admission [5], with even modest rates of misclassification translating into hundreds of thousands of dollars in unnecessary expenditures. In our population, if accurate identification and reclassification of pre-existing thrombi cases had been correctly classified as present on admission (POA), the estimated cost savings would have ranged from $714,000 to $1,000,000. Beyond financial implications, inflated PSI-12 rates may lead to unwarranted quality investigations and misdirect hospital resources. This highlights the urgent need for hospitals to refine PSI-12 classification criteria and ensure accurate documentation to prevent unnecessary financial penalties.

At our institution, we have implemented a collaborative effort between clinical documentation integrity teams and physicians to conduct weekly clinical reviews of PSI-12 events. This initiative has led to a significant reduction in PSI-12 rates by ensuring that thrombi identified on early duplex imaging are appropriately classified as POA when applicable. Expanding similar programs at other institutions could mitigate the impact of PSI-12 misclassification and improve the accuracy of quality metrics used for hospital performance assessment.

One important consideration in this study is the concept of “the more you look, the more you find.” As the use of early duplex studies increases, more clinically insignificant DVTs or thrombi of unknown chronicity may be detected. Current guidelines are not well-defined on how to manage these incidental findings, particularly when their clinical significance is unclear. Management should therefore be individualized, considering factors such as patient risk profile, surgical urgency, and thrombus characteristics. Treatment options may include delayed surgery with anticoagulation, placement of an inferior vena cava (IVC) filter, or proceeding with surgery with close postoperative monitoring. Further studies are needed to establish standardized protocols for incidental DVT management in preoperative patients.

This study has several limitations. First, its retrospective design may introduce selection bias, particularly in how PSI-12 events were recorded and reviewed. Second, while early duplex studies were performed within 48 h of admission, this timeframe does not necessarily confirm that thrombi were pre-existing, as some duplexes were obtained postoperatively and may reflect hospital-acquired thrombi. Third, this cohort included only patients who underwent early duplex studies, meaning it likely represents a higher-risk population with a higher prevalence of disease compared to the general surgical population. Finally, as this was conducted at a single institution, the findings may not be generalizable to all hospitals, particularly those with different patient populations, surgical case mixes, or PSI-12 reporting practices.

Our findings suggest that early duplex ultrasound screening in select patients can identify potentially pre-existing DVTs, and that PSI-12 misclassification is both common and carries significant implications for hospital quality metrics and finances. Incorporating admission venous duplex studies into PSI-12 evaluations could provide a more accurate assessment of thrombus timing and etiology, thus reducing misclassification rates and improving patient safety. As hospitals continue to refine quality improvement strategies, integrating early duplex screening, enhanced documentation review processes, and standardized classification criteria may help ensure that PSI-12 remains a valid and meaningful measure of surgical quality. Future multi-institutional studies are needed to validate these findings and refine standardized approaches to perioperative VTE surveillance.

## Conclusions

Our findings suggest that a substantial proportion of PSI-12 positive venous thromboembolism events may represent pre-existing disease rather than truly hospital-acquired VTE. One third of PSI-12 positive patients had abnormal early duplex findings, and nearly 9% of cases were misclassified, highlighting important limitations of PSI-12 as a surgical quality metric. Incorporating admission duplex studies in selected high-risk patients may improve the accuracy of PSI-12 classification, reduce unnecessary healthcare costs, and ensure that patient safety metrics more accurately reflect preventable harm.

## Data Availability

The datasets used are not publicly available as they originate from an institutional quality improvement database but are available from the corresponding author on reasonable request and with appropriate institutional approvals.

## Abbreviations

VTE: Venous thromboembolism
DVT: Deep vein thrombosis
PE: Pulmonary embolism
AHRQ: Agency for Healthcare Research and Quality
PSI: Patient Safety Indicator
POA: Present on Admission
VQI: Vascular Quality Initiative
NSQIP: National Surgical Quality Improvement Program
